# RPE vs. Percentage 1RM Loading in Periodized Programs Matched for Sets and Repetitions

**DOI:** 10.3389/fphys.2018.00247

**Published:** 2018-03-21

**Authors:** Eric R. Helms, Ryan K. Byrnes, Daniel M. Cooke, Michael H. Haischer, Joseph P. Carzoli, Trevor K. Johnson, Matthew R. Cross, John B. Cronin, Adam G. Storey, Michael C. Zourdos

**Affiliations:** ^1^Sport Performance Research Institute New Zealand, Auckland University of Technology, Auckland, New Zealand; ^2^Muscle Physiology Laboratory, Department of Exercise Science and Health Promotion, Florida Atlantic University, Boca Raton, FL, United States; ^3^School of Exercise, Biomedical and Health Sciences, Edith Cowan University, Perth, WA, Australia

**Keywords:** perceived exertion, resistance training, strength, autoregulation, powerlifting

## Abstract

**Purpose:** To investigate differences between rating of perceived exertion (RPE) and percentage one-repetition maximum (1RM) load assignment in resistance-trained males (19–35 years) performing protocols with matched sets and repetitions differentiated by load-assignment.

**Methods:** Participants performed squats then bench press 3x/weeks in a daily undulating format over 8-weeks. Participants were counterbalanced by pre-test 1RM then assigned to percentage 1RM (1RMG, *n* = 11); load-assignment via percentage 1RMs, or RPE groups (RPEG, *n* = 10); participant-selected loads to reach target RPE ranges. Ultrasonography determined pre and post-test pectoralis (PMT), and vastus lateralis muscle thickness at 50 (VLMT50) and 70% (VLMT70) femur-length.

**Results:** Bench press (1RMG +9.64 ± 5.36; RPEG + 10.70 ± 3.30 kg), squat (1RMG + 13.91 ± 5.89; RPEG + 17.05 ± 5.44 kg) and their combined-total 1RMs (1RMG + 23.55 ± 10.38; RPEG + 27.75 ± 7.94 kg) increased (*p* < 0.05) in both groups as did PMT (1RMG + 1.59 ± 1.33; RPEG +1.90 ± 1.91 mm), VLMT50 (1RMG +2.13 ± 1.95; RPEG + 1.85 ± 1.97 mm) and VLMT70 (1RMG + 2.40 ± 2.22; RPEG + 2.31 ± 2.27 mm). Between-group differences were non-significant (*p* > 0.05). Magnitude-based inferences revealed 79, 57, and 72% chances of mean small effect size (ES) advantages for squat; ES 90% confidence limits (CL) = 0.50 ± 0.63, bench press; ES 90% *CL* = 0.28 ± 0.73, and combined-total; ES 90% *CL* = 0.48 ± 0.68 respectively, in RPEG. There were 4, 14, and 6% chances 1RMG had a strength advantage of the same magnitude, and 18, 29, and 22% chances, respectively of trivial differences between groups.

**Conclusions:** Both loading-types are effective. However, RPE-based loading may provide a small 1RM strength advantage in a majority of individuals.

## Introduction

The principle of individualization is paramount to consider in the design of resistance training protocols to optimize adaptations (Borresen and Ian Lambert, 2009; Kiely, 2012). Indeed, evidence exists demonstrating that training adaptation is improved when program design is tailored to the athlete (Beaven et al., 2008a,b; Jones et al., 2016). One method of individualizing resistance training is “autoregulating” load prescription through the use of a rating of perceived exertion (RPE) (Helms et al., 2016).

Recently, an iteration of the traditional RPE scale based on “repetitions in reserve” (RIR) prior to muscular failure at the end of a set, was introduced to the literature (Zourdos et al., 2016). The RIR-based RPE scale may have more utility compared to traditional Borg RPE, which has yielded submaximal scores (6.8–9.0) even when an individual performs a set to volitional failure (Shimano et al., 2006; Pritchett et al., 2009; Hackett et al., 2012). Therefore, it has been recently suggested RIR-based RPE is superior to traditional RPE for assessing intensity during resistance training (Helms et al., 2016). Additionally, researchers have reported males and females to determine RIR accurately (within ~1 repetition) during the leg and chest press exercises when sets are performed within 0–3 repetitions from failure (Hackett et al., 2017). In further support of the RIR-based RPE approach, scores have been strongly and inversely correlated with velocity for both the squat (*r* = −0.87, *p* < 0.001) and bench press (*r* = −0.79, *p* < 0.001; Helms et al., 2017b), the implication being that as movement velocity decreases with higher intensities, reported RPE increases (RIR decreases).

Despite recent research regarding RIR-based RPE and the importance of individualization in resistance training prescription, training load is commonly prescribed as a percentage of pre-test one-repetition maximum (1RM) (Fleck and Kraemer, 2014). However, if atypical performance occurred during the pre-test 1RM or if there were testing administration errors, loading based on percentage 1RM could then lead to an inappropriate stimulus during training (Zourdos et al., 2016). Furthermore, the number of repetitions which can be performed at the same percentage of 1RM can differ substantially between athletes based on genetic differences and training background (Richens and Cleather, 2014). Thus, various issues exist when prescribing load solely with percentage of 1RM, whereas RPE can account for individual differences in repetitions allowed and rate of adaptation. However, to our knowledge there is no study which has compared changes in strength and hypertrophy over time between percentage based and RPE based training programs.

Therefore, the purpose of this study was to compare two resistance training protocols with matched repetitions, sets, exercises, and rest periods, but with differing methods of load prescription; one group using percentage of pre-test 1RM and the other using the RIR-based RPE scale. We hypothesized the method of load prescription would create minimal differences in total volume (sets × repetitions × load) between groups and likewise, minimal differences in hypertrophy (Schoenfeld et al., 2014). However, we hypothesized that intensity (both RPE and percentage of pre-test 1RM) would differ between groups, and that the RPE group would increase strength to a greater extent than the percentage-based group due to load progression aligning more closely to each individual participant's capabilities (Klemp et al., 2016).

## Materials and methods

### Participants

A total of 24 males began this study. Three participants dropped out, two due to minor injury (joint pain or muscular discomfort) and one due to a family emergency; therefore, 21 participants completed the protocol (Table 1). Inclusion criteria were as follows: (1) minimum resistance training experience of 2 years while also performing the back squat and bench press a minimum of once per week for the last 6 months; (2) a minimum 1RM back squat and bench press of 1.5x and 1.25x body mass, respectively; and (3) be free from injury/illness that would contraindicate participation. Resistance training history was determined by completing a questionnaire previously used with similar populations (Klemp et al., 2016). All participants were informed of potential risks and signed an informed consent document prior to participation. Ethics approval was granted by the University Institutional Review Board.

**Table 1:** Descriptive characteristics of participants.

**Variable**	**1RMG (*n* = 11 males)**	**RPEG (*n* = 10 males)**	**Combined (*n* = 21 males)**
Height (m)	1.75 ± 0.08	1.72 ± 0.06	1.74 ± 0.07
Body Mass (kg)	80.2 ± 12.2	78.8 ± 9.72	79.5 ± 10.8
Body Fat (%)	10.8 ± 6.1	11.4 ± 5.1	11.1 ± 5.5
Age (yrs)	23.8 ± 4.2	20.9 ± 1.4	22.4 ± 3.4
PMT (mm)	28.5 ± 6.4	30.6 ± 6.5	29.5 ± 6.4
VLMT50 (mm)	27.9 ± 3.6	27.3 ± 4.5	27.6 ± 4.0
VLMT70 (mm)	24.2 ± 3.1	23.8 ± 3.4	24.0 ± 3.2
Squat 1RM (kg)	139.2 ± 18.2	143.7 ± 24.9	141.3 ± 21.2
Bench Press 1RM (kg)	113.9 ± 18.7	120.9 ± 19.3	117.2 ± 18.8
Squat Wilks Score	96.6 ± 15.0	99.4 ± 11.9	98.0 ± 13.3
Bench Press Wilks Score	78.0 ± 7.9	83.8 ± 9.5	80.8 ± 8.9

*1RMG, percentage 1RM load group; RPEG, RPE load group. Values are mean ± standard deviation. 1RM, one repetition maximum; PMT, pectoralis major muscle thickness; VLMT50, vastus lateralis muscle thickness at 50% femur length; VLMT70, vastus lateralis muscle thickness at 50% femur length. Combined, mean values of all 21 participants. Wilks score (Vanderburgh and Batterham, 1999) is the scoring system used in powerlifting to determine strength relative to body mass*.

### Experimental design

The aim of this study was to compare strength and hypertrophy adaptations in trained individuals following a daily undulating periodization model, differentiated only by load prescription (RPE or percentage of 1RM). Groups were counterbalanced to ensure minimal differences (mean 1RMs as similar as possible with as high a *p*-value as possible when comparing means) in absolute and relative 1RM strength as measured by the Wilks score (a validated method of measuring relative strength in competitive powerlifting) (Vanderburgh and Batterham, 1999). Participants were assigned to either a percentage 1RM group (1RMG, *n* = 11) with load assigned as percentages of pre-test 1RMs, or to an RPE group (RPEG, *n* = 10) with load selected by participants to reach target RPE ranges.

A training duration of 8 weeks, while following a daily undulating periodized model, was selected as significant 1RM and muscle thickness increases were recently reported in a study of this length on a similarly sized and trained population of males following similar progressions in volume and intensity (Klemp et al., 2016). Exercise selection, rest periods, and prescribed set and repetitions were identical among groups. Both groups trained 3 times/week on non-consecutive days (i.e., Monday, Wednesday, and Friday) and performed the specified repetitions in a fixed, descending order each week. In a linear format, every 2 weeks (after the introductory week) the prescribed repetitions decreased as load (either RPE or percentage of 1RM) increased throughout. The final week consisted of a lowered volume taper leading into post-testing on the final day. The specific details of the programs' structure are outlined in Table 2.

**Table 2:** Summary of training plans.

	**Percentage 1RM group (1RMG)**			**RPE group (RPEG)**		
**Week**	**Monday**	**Wednesday**	**Friday**	**Monday**	**Wednesday**	**Friday**
0	x	x	1RM Testing	x	x	1RM Testing
1	2 × 8 × 65%	2 × 6 × 70%	2 × 4 × 75%	2 × 8 × 5–7 RPE	2 × 6 × 5–7 RPE	2 × 4 × 5–7 RPE
2	3 × 8 × 70%	3 × 6 × 75%	3 × 4 × 80%	3 × 8 × 6–8 RPE	3 × 6 × 6–8 RPE	3 × 4 × 6–8 RPE
3	3 × 8 × 72.5%^*^	3 × 6 × 77.5%^*^	3 × 4 × 82.5%^*^	3 × 8 × 6–8 RPE	3 × 6 × 6–8 RPE	3 × 4 × 6–8 RPE
4	3 × 7 × 75%	3 × 5 × 80%	3 × 3 × 85%	3 × 7 × 7–9 RPE	3 × 5 × 7–9 RPE	3 × 3 × 7–9 RPE
5	3 × 7 × 77.5%^*^	3 × 5 × 82.5%^*^	3 × 3 × 87.5%^*^	3 × 7 × 7–9 RPE	3 × 5 × 7–9 RPE	3 × 3 × 7–9 RPE
6	3 × 6 × 80%	3 × 4 × 85%	3 × 2 × 90%	3 × 6 × 8–10 RPE	3 × 4 × 8–10 RPE	3 × 2 × 8–10 RPE
7	3 × 6 × 82.5%^*^	3 × 4 × 87.5%^*^	3 × 2 × 92.5%^*^	3 × 6 × 8–10 RPE	3 × 4 × 8–10 RPE	3 × 2 × 8–10 RPE
8	2 × 4 × 80%	2 × 3 × 85%	1RM Testing	2 × 4 × 6–8 RPE	2 × 3 × 6–8 RPE	1RM Testing

*1RMG uses percentages of pre-test 1RM to assign loads while RPEG uses RPE based on repetitions in reserve. Values are displayed as sets × repetitions × load*.

* *If all repetitions were completed with previous week's assigned loads, load was increased as listed. If any repetitions are missed, load remained the same as prior week. 1RM, one repetition maximum; RPE, rating of perceived exertion*.

### One-repetition maximum (1RM) testing

Participants were shown the resistance-training specific RPE scale while receiving verbal instruction on how scores are determined (Zourdos et al., 2016). Briefly, this 1–10 rating scale is based on the subjective determination of RIR prior to reaching failure such that a score of 10 indicates the maximal number of repetitions for that load were performed, 9.5 indicates no further RIR but the same number of repetitions could be performed with a slightly heavier load, 9 indicates one RIR, 8.5 indicates 1–2 RIR, 8 indicates 2 RIR, etc. Following this explanation, participants performed a standardized, bodyweight, dynamic warm-up and then according to previously validated procedures (Zourdos et al., 2016), the investigators proceeded to test the 1RM of their back squat, followed by their bench press. To aid the researchers in attempt selection, average concentric velocity (ACV) using a Tendo Weightlifting Analyzer (TENDO Sports Machines, Trencin, Slovak Republic), and RPE scores were collected after the final warm-up and each 1RM attempt (Zourdos et al., 2016). Previously researchers have identified that comparably trained lifters approached an ACV of ~0.20 m·s^−1^ and ~0.15 m·s^−1^ on average for the squat and bench press, respectively, at 1RM (Helms et al., 2017b; Ormsbee et al., 2017). Thus, the investigators made smaller increases in load for 1RM attempts as velocity neared these thresholds. Additionally, during post-testing, the velocity at which pre-test 1RMs were recorded was used to gauge when a participant was approaching 1RM. Likewise, the proximity to this velocity was used to aid 1RM post-test attempt selection. To provide a clear standard for the parameters of form, both exercises were performed in accordance with the standards of the International Powerlifting Federation (IPF, 2016) and a National Strength and Conditioning Association certified strength and conditioning specialist with experience coaching powerlifters monitored all testing and training sessions. Barbells and weight plates were calibrated (Eleiko Sport, Korsvägen, Halmstad, Sweden), and fractional plates (to the nearest 0.25 kg) were used to ensure loading precision in all testing sessions.

### Training protocol

While the RPEG self-selected their loads to reach the target RPE range, both groups provided RPE scores after their final warm-up set and all working sets to allow RPE comparisons between groups throughout the study. Percentage 1RM assignments in 1RMG were based upon recently published loading relationships in an attempt to ensure that, on average, the assigned percentages in 1RMG would fall within the corresponding RPE ranges assigned to RPEG. As an example, in our previously published work trained lifters reported a 7 RPE when performing eight repetitions with 70% of 1RM (Helms et al., 2016). So, in week 2 day 1, when 1RMG performed eight repetitions with 70% of 1RM, the RPEG performed eight repetitions with a load that resulted in a 6–8 RPE (Table 2). Participants reported to the laboratory to perform monitored resistance training for a total of 25 days over 8 consecutive weeks. Each training session took place at the same time each day to account for any diurnal changes in strength. Pre- and post-testing for anthropometric measurements, muscle thickness, and 1RM strength took place 48–72 h before week 1 and at the end of week 8, respectively. After pre-testing, participants returned to the lab 48–72 h later to begin a lower volume and load introductory microcycle during week 1 (Table 2). The “main training program” occurred in weeks 2–7, then during week 8 participants completed taper sessions on the first 2 days of training, and post-testing occurred on the final day of the taper and test week.

In 1RMG load was assigned as a percentage of pre-test 1RM and progressed in a linear fashion throughout the study. However, on weeks 3, 5, and 7, load was only increased by 2.5% of 1RM if all sets and repetitions were completed on the same day from the prior week. If any repetitions were not able to be completed from the prior week, load remained the same (Table 2). Within-week, if a participant was unable to complete repetitions, the load was reduced 4% for every repetition missed on the subsequent set for the same exercise. During week 1 for the RPEG, the researchers selected loads for the participants to ensure the goal of the introductory week was accomplished (acclimating the participants to the frequency and total volume of training) and to aid in familiarizing the participants with RPE-based load selection. The researchers explained their rationale for load selection to the participants during week 1 to better familiarize the participants for weeks 2–8 where they self-selected load. Investigators selected the loads during week 1 based on the combined factors of the percentage of 1RM they expected to fall within the RPE range, the RPE of the last warm-up set and visual assessment of barbell speed. Additionally, researchers conservatively estimated loads to land at the lower end of the target RPE range to prevent cumulative fatigue from pushing the subsequent set above the RPE range.

In weeks 2–8, RPEG participants were shown the record of their performance on the same day of the previous week to assist them in daily load selection. In all weeks, when the reported RPE score for a completed set fell outside of the target RPE range, an automatic adjustment to the load was made for the subsequent set. Based on previous research (Helms et al., 2017a), for every 0.5 RPE above or below the upper or lower RPE threshold, respectively, load was decreased or increased by 2% in an attempt to bring the subsequent set's RPE closer to the assigned range. An example of how this load adjustment protocol was implemented for an RPE range of 6–8 is displayed in Table 3. When the load fell within the assigned RPE range, the participant (or the researchers in the case of week 1) had the choice to modify load as desired so long as they believed it would still fall within the target RPE range. If a participant missed assigned repetitions, for example completing 7 repetitions when 8 were assigned, the set was considered a 10 RPE and each missed repetition was considered a full RPE point for load-adjustment purposes (i.e., if 5 repetitions at a 7–9 RPE was assigned, and 4 repetitions were completed, the load on the subsequent set would be reduced by 8%; 4% for being a full RPE point above the upper threshold of the range and an additional 4% for being 1 repetition short of the target). In both groups, 5–7 min rest periods were administered between working sets and after the final warm up set before the first working set. Additionally, the squat was performed prior to the bench press and a 10 min rest period occurred after concluding the squat prior to initiating the bench press.

**Table 3:** Example RPE load adjustments.

**Actual RPE**	**Assigned RPE range 6–8**
1	Increase load by 20%
2	Increase load by 16%
3	Increase load by 12%
4	Increase load by 8%
5	Increase load by 4%
6	Participant choice
7	Participant choice
7.5	Participant choice
8	Participant choice
8.5	Decrease load by 2%
9	Decrease load by 4%
9.5	Decrease load by 6%
10	Decrease load by 8%

*RPE, rating of perceived exertion*.

### Dietary logs, protein, and amino acid provision

To encourage consistent energy and food intake throughout training and testing, a 3 consecutive-day food log was completed during the first week of training and then again during the final week. In the interim period and prior to the final week food log, participants were instructed to continue their normal dietary habits. To control for the potential impact of nutrient timing between groups, participants ingested branched chain amino acids (Xtend, Scivation, Burlington, N.C., USA) containing 3.5 g of leucine approximately 20 min prior to each training and testing session (upon arriving at the lab, then they began their dynamic warm up 10 min after) and 30 g of whey protein (Scivation Whey, Scivation, Burlington, N.C., USA) immediately after each session. Both whey protein and branched-chain amino acids were provided because of their ability to enhance muscle protein synthesis (Tipton et al., 2001; Moore et al., 2009).

### Muscle thickness testing

Pectoralis major muscle thickness (PMT) and 50% (VLMT50) and 70% vastus lateralis muscle thickness (VLMT70) were assessed via ultrasonography (Bodymetrix Pro System, Intelemetrix Inc., Livermore, Calif., USA) prior to 1RM pre and post-testing. This method of testing was previously used to assess the growth response to resistance training (Schoenfeld et al., 2014) and was validated with magnetic resonance imaging (Reeves et al., 2004). Scans were performed prior to 1RM assessment on the right side of the body during pre- and post-testing. Sites were scanned lateral to medial with the transducer perpendicular to the skin. Sites were scanned twice and an average of the two scans was recorded. However, if the difference between the two scans was >2 mm, a third was performed and the two values within 2 mm were averaged. The site for the chest was designated as half the distance between the nipple and the anterior axillary line. Vastus lateralis scans were performed in the supine position. Sites were marked and measured at 50 and 70%, respectively, of the distance from the greater trochanter to the lateral epicondyle of the femur (Abe et al., 1994, 1998). All scans were performed by the same investigator.

### Readiness questionnaires

Prior to beginning warm up sets, participants completed part A and B of the daily analysis of life demands for athletes (DALDA) questionnaire and recorded a 1–10 perceived recovery status (PRS) score by hand. The DALDA is a two-part questionnaire consisting of an A, B, or C Likert scale in which users record whether they (A), feel worse than normal, (B), feel normal, or (C), feel better than normal. Part A consists of 9 broad categories in which stress can be assessed and part B consists of a list of 25 questions pertaining to specific sources of stress (Rushall, 1990). The PRS scale is a simple 0–10 scoring system where the higher the score indicates greater recovery and the more likely the individual would expect improved performance (Laurent et al., 2011).

### Statistical analysis

To assess within group pre to post changes in muscle thickness and strength, we performed independent paired *T*-tests set at an alpha of 0.05. Despite relative homogeneity due to counterbalancing, there was still some variation between groups in 1RM strength and muscle thickness. Thus, to analyse differences between groups we utilized analyses of covariance with pre-test scores as covariates. This is the preferred method of analysis to account for the fact that participants with low pre-test scores generally improve more than those with high pre-test scores (Vickers and Altman, 2001).

To supplement null hypothesis testing, we calculated between group effect size (ES) values such that each groups' change score (post-test–pre-test) was divided by the pooled standard deviation (SD) of both groups' change scores (Morris and DeShon, 2002; Page, 2014; Dankel et al., 2017). Thresholds for ES were based on Hopkins' scale such that an ES of < 0.20 was considered trivial, and threshold values of 0.20, 0.60, 1.20, and 2.00 were used to represent small (and the smallest worthwhile effect), moderate, large, and very large effects (Batterham and Hopkins, 2006; Hopkins et al., 2009). Additionally, we calculated the 90% confidence limits (CL) of each ES, using the small sample size bias adjustment of the SD outlined by Becker (Becker, 1988; Morris, 2008), to determine the probability that there was a positive (≥ 0.20 ES), trivial (0.19 to −0.19 ES), or negative (≤ −0.20 ES) effect of the “intervention” (RPEG). Based on the same rationale for utilizing an analysis of covariance, we used the Hopkins spreadsheet “analysis of a pre-post parallel-groups controlled trial with adjustment for a predictor” (Hopkins, 2006) with the pre-test values as the covariate for the above calculations. For clarity of interpretation, rather than presenting the likelihood of a negative effect of the “intervention” (RPEG) relative to the “control” (1RMG) with negative ES values, we removed the sign and presented this as the probability of an advantage of the 1RMG. Thus, data is presented as the probability of an advantage of RPEG, 1RMG or a trivial difference between groups.

Finally, differences between groups for the mean total across the 8-week study and at each time point (weeks 1–8) for the average weekly RPE, relative volume load (sets × repetitions × percentage 1RM), relative intensity per repetition (average percentage 1RM per repetition for the week), change in PRS and change in DALDA scores were determined by 2 tailed independent *T*-tests with an alpha of 0.05. Analyses were performed using a statistical software package (IBM SPSS Statistics 21, SPSS Inc., Chicago, IL).

## Results

### Participant adherence

Participants were required to complete at least 90% of all sessions to be included (no more than two missed sessions and no missed sessions during the taper). The 1RMG as a whole completed 98% of all sessions. The RPEG as a whole completed 97% of the squat portion and 96.5% of the bench portion of the sessions (in one instance a participant did not bench press as a precaution due to shoulder discomfort, which subsided by the next training session).

### One-repetition maximum (1RM) strength and muscle thickness

Both 1RMG and RPEG significantly increased back squat, bench press, and combined 1RM strength relative to baseline (*p* < 0.001). Specifically, squat 1RM increased in 1RMG by 13.9 ± 5.9 kg and in RPEG by 17.1 ± 5.4 kg while bench press 1RM increased by 9.6 ± 5.4 kg and 10.7 ± 3.3 kg in 1RMG and RPEG, respectively. Post-test 1RM back squat was 153.1 ± 16.6 and 160.7 ± 28.4 and post-test 1RM bench press was 113.9 ± 18.7 and 131.6 ± 19.5 in 1RMG and RPEG, respectively. Combined squat and bench press 1RM increased by 23.6 ± 10.4 kg in 1RMG and by 27.8 ± 7.9 kg in RPEG.

Additionally, muscle thickness significantly increased at all measurement sites in both groups relative to baseline. Specifically, PMT increased in 1RMG by 1.6 ± 1.3 mm (*p* < 0.001) and in RPEG by 1.9 ± 1.9 mm (*p* < 0.001). Post-test PMT was 30.1 ± 6.7 and 32.5 ± 6.8 in 1RMG and RPEG, respectively. Likewise, VLMT50 increased by 2.1 ± 2.0 mm (*p* = 0.004) and 1.9 ± 2.0 mm (*p* = 0.01) in 1RMG and RPEG, respectively. Post-test VLMT50 was 30.0 ± 4.2 and 29.1 ± 4.9 in 1RMG and RPEG, respectively. Finally, VLMT70 increased in 1RMG by 2.4 ± 2.2 mm (*p* = 0.004) and in RPEG by 2.3 ± 2.3 mm (*p* = 0.02). Post-test VLMT70 was 26.6 ± 4.0 and 26.1 ± 2.7 in 1RMG and RPEG, respectively.

Overall, there were no significant differences observed between groups for 1RM or muscle thickness. However, there were small between group ES for squat, bench press, and combined 1RM which all favored RPEG. Exact *p* values and the ES 90% CL, along with probabilities of advantage or trivial difference are displayed in Table 4.

**Table 4:** Strength and muscle thickness changes.

**Variable**	***P*-value**	**Size of effect (mean ± 90% CL)**	**Chance of RPE-loading advantage (≥0.20 ES) (%)**	**Chance of trivial difference (−0.19 to 0.19 ES) (%)**	**Chance of %1RM-loading advantage (≥0.20 ES) (%)**
Squat 1RM	0.32	0.50 ± 0.63	79	18	4
Bench 1RM	0.52	0.28 ± 0.73	57	29	14
Combined 1RM	0.38	0.48 ± 0.68	72	22	6
PMT	0.66	0.15 ± 0.79	46	32	22
VLMT50	0.76	−0.13 ± 0.76	23	33	44
VLMT70	0.79	−0.06 ± 0.68	25	38	37

*Between group differences in strength and muscle thickness. CL, confidence limit; RPE, rating of perceived exertion; ES, effect size; 1RM, one repetition maximum; PMT, pectoralis major muscle thickness; VLMT50, vastus lateralis muscle thickness at 50% femur length; VLMT70, vastus lateralis muscle thickness at 50% femur length*.

### Training RPE, volume, and intensity

**Figure 1:**
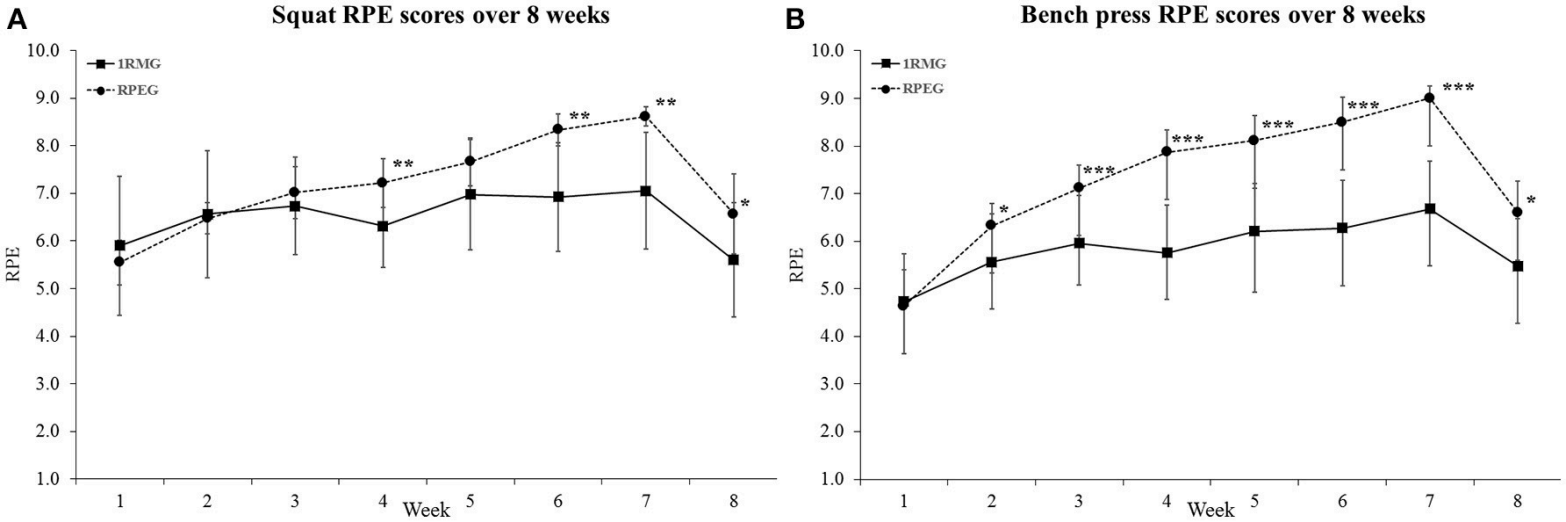
Weekly average RPE values for **(A)** for squat and **(B)** bench press. ^*^p< 0.05, ^**^p < 0.01, and ^***^p < 0.001. RPE, rating of perceived exertion.

For the squat, RPE was significantly higher in RPEG vs. 1RMG in weeks 4, 6, 7, and 8, and the difference approached significance (*p* = 0.09) in week 5. Likewise, RPE was higher for the bench press in RPEG during weeks 2–8 compared to 1RMG. Figure 1 displays the weekly average RPE scores for both groups, for both lifts, throughout the study. Average squat RPE for the entire 8-week period also significantly differed (*p* = 0.04) with higher values in RPEG (7.2 ± 0.3) compared to 1RMG (6.5 ± 1.0). Likewise, average bench press RPE for the 8-week period was significantly (*p* < 0.001) higher in RPEG (7.3 ± 0.3) compared to 1RMG (5.8 ± 1.0).

**Figure 2:**
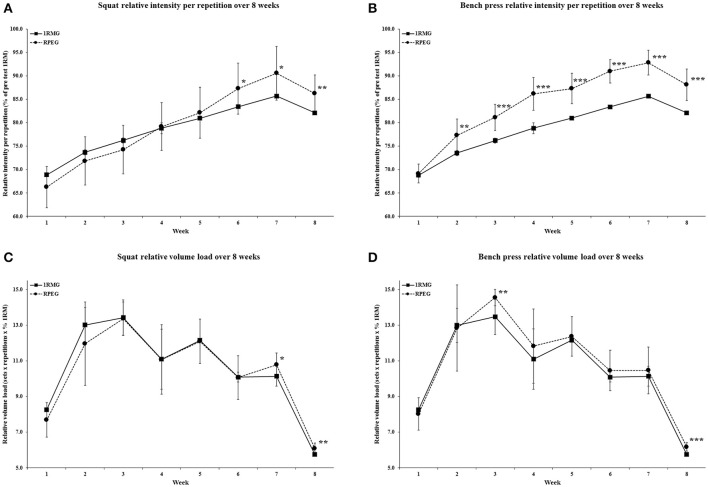
Weekly average values for **(A)** intensity relative to pre-test 1RM per repetition for squat, **(B)** bench press and **(C)** volume load relative to pre-test 1RM for squat and **(D)** bench press. ^*^p < 0.05, ^**^p < 0.01, and ^***^p < 0.001. 1RM, one repetition maximum.

Similarly, weekly average relative intensity per repetition (defined as the load used in training divided by the pre-test 1RM) diverged with significantly higher values in RPEG at weeks 6–8 and 2–8 in the squat and bench press respectively, compared to 1RMG. As 1RMG had a pre-planned load, changes in relative intensity per repetition illustrate how RPEG increased training loads throughout the study, comparatively. Lastly, relative volume load differed significantly between groups with RPEG performing more volume than 1RMG at weeks 7 and 8 and weeks 3 and 8 for the squat and bench press, respectively. The relative intensity per repetition and relative volume load values for both groups, for both lifts, throughout the study are displayed in Figure 2.

Average relative intensity for the entire 8-week period was not significantly different between 1RMG (78.73 ± 0.20%) and RPEG (79.73 ± 4.44%) for squat (*p* = 0.49). Likewise, average relative volume load for the entire 8-week period was not significantly different between 1RMG (10.49 ± 0.21) and RPEG (10.39 ± 0.67) for squat (*p* = 0.66). However, average relative intensity for the entire 8-week period was significantly greater in RPEG (84.14 ± 2.02%) compared to 1RMG (78.70 ± 0.18%) for bench press (*p* < 0.001). Additionally, average relative volume load for the entire 8-week period was also significantly greater in RPEG (10.84 ± 0.41) compared to 1RMG (10.49 ± 0.21) for bench press (*p* = 0.03).

### Perceived readiness

Week to week changes in DALDA part A, part B and PRS scores were not significantly different between groups at any time point (data not shown). However, the change in average PRS score from week 6 to 7 in RPEG (−0.6 ± 0.5) vs. 1RMG (−0.1 ± 0.8) approached significance (*p* = 0.08). Likewise, the change from week 7 to 8 in RPEG (1.1 ± 1.1) vs. 1RMG (0.3 ± 1.0) also approached significance (*p* = 0.09).

## Discussion

The goal of this study was to compare two resistance training protocols differentiated only by loading strategy to determine if they would produce different muscle thickness, psychometric, and performance outcomes. Our first hypothesis that greater strength gains would be achieved by individualizing load assignment via RPE was partially supported. Null hypothesis testing did not reveal a significant difference between groups. However, small (0.28–0.50) between group ES differences were found with probabilities favoring RPEG. Our second hypothesis, that muscle thickness changes would be similar between groups was supported as there were no significant differences between 1RMG and RPEG for any muscle thickness measurement. Furthermore, between group ESs were trivial and probabilities were unclear.

Since the recent introduction of the RIR-based RPE scale to the literature (Zourdos et al., 2016), researchers have postulated that greater performance could be achieved by using the scale to “autoregulate” load (Helms et al., 2016, 2017b; Zourdos et al., 2016; Ormsbee et al., 2017). To our knowledge, this is the first study that has addressed and provided initial support for this claim. With that said, strength differences between groups were small and variable enough to fall short of statistical significance. This may indicate that while some individuals could benefit from using RPE as a loading strategy, for others, the choice between using percentage 1RM- or RPE-based loading is inconsequential (at least in the short term). However, at the group level the RPEG trained at a higher average RPE than 1RMG. Specifically, RPE diverged at week 4 for squat and week 2 for bench press and then remained higher in RPEG throughout the rest of the study. Interestingly, significant increases in strength and hypertrophy occurred in both groups, despite the majority of training occurring ~3–4 repetitions from failure (RPE ~6–7). This provides further evidence that training to failure at all times is not necessary to make significant gains in hypertrophy (Sampson and Groeller, 2016) or strength (Izquierdo et al., 2006; Davies et al., 2016), at least when training with moderate to heavy loads (Ogasawara et al., 2013).

Mirroring this divergence in RPE, relative intensity per repetition was also higher beginning at week 6 for squats and week 2 for bench press in RPEG compared to 1RMG. Thus, for a large part of the study RPEG trained at a higher RPE and percentage of pre-test 1RM than 1RMG, which may explain the higher probability of enhanced 1RM in RPEG. Differences in relative volume load were not expected since we matched sets and repetitions. Nonetheless, likely due to the higher relative intensity (as relative volume is sets × repetitions × percentage 1RM), RPEG performed more bench press volume overall and more volume at two time points for the squat (weeks 7 and 8) and bench press (weeks 3 and 8). Related to the volume performed, our second hypothesis that muscle thickness changes would be similar between groups, was supported. As stated, while there were some differences in volume performed between groups, it was not substantial enough to generate greater hypertrophy in the short-term.

Interestingly, the PRS changes between groups approached significance (*p* = 0.08–0.09) at weeks 7 and 8. The RPEG had a larger decrease in PRS from week 6 to 7 and then a larger increase in PRS from week 7 to 8, compared to 1RMG. This might indicate that in the final week prior to the taper where load was the highest (week 7), RPEG overreached to a greater extent than 1RMG and that the taper was more effective for RPEG, as their PRS score rebounded to a greater degree during week 8. This PRS score pattern provides some insight into how RPE-based loading may help to ensure the temporal goals of a mesocycle are adhered to. On the other hand, changes in DALDA scores between groups were non-significant at all time points. However, based on our anecdotal observation of the participants, as time went on the DALDA forms were completed more quickly, with less effort and with less attention to detail. This might highlight a potential advantage of the PRS compared to the DALDA, in that it takes less effort and time to evaluate readiness using a singular 1–10 scale compared to a 34 item, 3 point Likert-scale questionnaire.

A limitation of this study is that strength improvement may have been greater in RPEG because the prescribed percentages of 1RM were too low or the progression rate was too slow in 1RMG, whereas participants in RPEG were able to progress at an individualized rate. While we made an effort to assign percentages of 1RM which should yield similar RPE to the range prescribed in RPEG (Helms et al., 2016), greater total volume (*p* = 0.03) at a higher average intensity (*p* < 0.001) was performed by RPEG for the bench press. However, the relevance of this difference is questionable, as there were not significant differences between groups for the squat in total volume (*p* = 0.49) or average intensity per repetition (*p* = 0.66), yet the squat had the highest probability of greater strength gain due to RPE-based loading. Alternatively, if this is a limitation of the study, it might also be a limitation of percentage 1RM-based loading in general, as the number of “repetitions allowed” at a given percentage of 1RM and rates of adaptation differ substantially between individuals (Richens and Cleather, 2014).

In summary, both 1RMG and RPEG increased 1RM squat and bench press (*p* < 0.001) along with both upper and lower body muscle thickness (*p* < 0.05) over the course of 8 weeks. Although no statistically significant differences between groups existed, there were small between-group ESs in favor of RPEG for 1RM squat (0.50) and bench press (0.28), which when analyzed probabilistically, translated to 79 and 57% greater odds for strength gain in favor of RPEG, respectively. Moreover, there were various points throughout the study where average RPE per set, relative volume and relative intensity per repetition were higher in RPEG vs. 1RMG, possibly explaining the likelihood of a small advantage in favor of RPEG for strength improvement.

Practically speaking, although RPEG may have provided a slight benefit in the present study for strength, this does not mean that RPE and percentage of 1RM should be seen as mutually exclusive for load prescription. For example, RPE accuracy may vary by individual; thus, a lifter who is inaccurate with RPE may not be advised to use solely RPE for load prescription. In this situation, a conservative percentage of 1RM can be assigned for a set number of repetitions for the initial set. However, a “goal” RPE range could also be established (i.e., 4 sets of 8 at 70% of 1RM with goal RPE of 6–8), and the individual could adjust the subsequent sets if the first set RPE is out of the goal range. The proposed strategy could also be used in a sports team setting where athletes with different training backgrounds and muscle characteristics may perform substantially different repetitions at the same percentage of 1RM (Richens and Cleather, 2014); thus, athletes could use the goal RPE range to adjust load accordingly. Furthermore, the strategy of using percentage 1RM and RPE in conjunction also accounts for daily readiness with a baseline structure, in that the individual has a pre-determined load, yet can adjust in accordance with the goal RPE if recovery between sessions was inadequate.

For future research, we recommend that inter-individual differences be explored. It has already been established that training age may impact the ability to accurately rate RPE (Zourdos et al., 2016). However, other characteristics such as temperament or social attitudes toward resistance training may influence RPE ratings and therefore could be used to predict which individuals might respond better to an RPE-based loading strategy.

## Ethics statement

This study was carried out in accordance with the recommendations of The Florida Atlantic University Institutional Review Board with written informed consent from all subjects. All subjects gave written informed consent in accordance with the Declaration of Helsinki. The protocol was approved by the The Florida Atlantic University Institutional Review Board.

## Author contributions

EH with the assistance of JBC, AS, and MZ developed the concept and overall design of the study. EH, RB, DC, MH, JPC, TJ, and MZ developed the specific data collection procedures, recruited the participants, and collected the data. RB conducted all ultrasound scans and conducted the raw ultrasound data analysis. RB, DC, and EH coordinated data collection sessions, times and schedules and contacted participants throughout the study. EH and MC performed the statistical analysis of the data. EH created the tables and figures and wrote the initial draft of the manuscript with the assistance of MC on the statistical analysis section. All authors revised and edited the manuscript.

### Conflict of interest statement

The authors declare that the research was conducted in the absence of any commercial or financial relationships that could be construed as a potential conflict of interest.
